# Humanized Mouse Models of Bacterial Infections

**DOI:** 10.3390/antibiotics13070640

**Published:** 2024-07-11

**Authors:** Katya McDonald, Adryiana Rodriguez, Gowrishankar Muthukrishnan

**Affiliations:** 1 Center for Musculoskeletal Research, Department of Orthopaedics, University of Rochester Medical Center, 601 Elmwood Avenue, Box 665, Rochester, NY 14642, USA; 2 Department of Microbiology and Immunology, University of Rochester Medical Center, Rochester, NY 14642, USA

**Keywords:** humanized mice, bacterial infections, *Staphylococcus aureus*, tuberculosis, *Mycobacterium tuberculosis*

## Abstract

Bacterial infections continue to represent a significant healthcare burden worldwide, causing considerable mortality and morbidity every year. The emergence of multidrug-resistant bacterial strains continues to rise, posing serious risks to controlling global disease outbreaks. To develop novel and more effective treatment and vaccination programs, there is a need for clinically relevant small animal models. Since multiple bacterial species have human-specific tropism for numerous virulence factors and toxins, conventional mouse models do not fully represent human disease. Several human disease characteristic phenotypes, such as lung granulomas in the case of *Mycobacterium tuberculosis* infections, are absent in standard mouse models. Alternatively, certain pathogens, such as *Salmonella enterica* serovar *typhi* and *Staphylococcus aureus*, can be well tolerated in mice and cleared quickly. To address this, multiple groups have developed humanized mouse models and observed enhanced susceptibility to infection and a more faithful recapitulation of human disease. In the last two decades, multiple humanized mouse models have been developed to attempt to recapitulate the human immune system in a small animal model. In this review, we first discuss the history of immunodeficient mice that has enabled the engraftment of human tissue and the engraftment methods currently used in the field. We then highlight how humanized mouse models successfully uncovered critical human immune responses to various bacterial infections, including *Salmonella enterica serovar Typhi*, *Mycobacterium tuberculosis*, and *Staphylococcus aureus*.

## 1. Introduction

The study of complex host–pathogen interactions to improve our knowledge of disease pathogenesis and design potential therapeutics necessitates the use of clinically relevant mouse models. This is true in the context of bacterial infections, which cause an astounding 7.7 million human deaths annually worldwide [1]. Murine models have greatly facilitated our understanding of bacterial pathogenesis due to the following reasons: (1) they are cost-effective, (2) the availability of genetics and molecular probes, and (3) they have similarities in their cardiovascular, nervous, and endocrine systems to humans [2,3,4]. However, they have several limitations, including altered diets and microbiomes, faster metabolic rates, and, most notably, altered innate and adaptive immune responses to bacterial infections, including differences in antibody isotypes produced, cell signaling pathways, and immune regulation [5,6,7,8]. In the last two decades, multiple humanized mouse models have been developed to attempt to recapitulate the human immune system in a small animal model [9]. There are two approaches to the concept of humanizing mice, of which the first is to create transgenic mice with a specific human gene (ex. PD-1 and VISTA) [10,11]. These models are used to investigate the impact of individual human proteins while the rest of the cells and proteins are murine. The second, the focus of this review, is the combination of immunodeficient mice and human cells and/or tissues. These humanized mouse models have been used extensively in the fields of cancer and viral infection, which have been comprehensively reviewed elsewhere [12,13,14,15]. In the last 15 years, investigators have also used these models to study bacterial pathogenesis and to specifically examine *Staphylococcus aureus*, *Salmonella enterica serovar typhi*, and *Mycobacterium tuberculosis*. This is due to the growing knowledge that bacterial pathogenesis is impacted by human-specific immune factors/proteins [16]. Therefore, the use of humanized mice can lead to a more extensive understanding of human host–pathogen interactions, which may inform future vaccines and therapeutics. This review aims to discuss the breadth of humanized mouse models used in bacterial infection research to highlight recent innovations in the field and their potential for use with other unexplored bacterial pathogens.

## 2. Immunodeficient Mice

The discovery and creation of immunodeficient mouse lines was critical to developing humanized mouse models. The first venture into the idea of immunodeficient mice was the discovery of nude mice in the 1960s, which have abnormal thymic development and, therefore, deficient T cells [17]. However, likely due to the presence of their other intact immune cells (functional B cells and myeloid cells), the engraftment of human immune cells was not feasible [18]. Next, severe combined immunodeficient (SCID) mice were discovered in the 1980s, which had a mutation in the *Prkdc* gene, leading to lymphocyte deficiency [19,20]. In these mice, labs were able to successfully engraft human peripheral blood leukocytes (PBLs) and demonstrate a human immune response to tetanus toxoid immunization and overall human lymphocyte survival and expansion [21]. However, the presence of NK cells and myeloid cells in these mice was linked to limited human immune cell survival in the engrafted animal [22]. Therefore, the SCID mice were crossed with nonobese diabetic (NOD/Lt) mice, which have defects in NK cells and antigen-presenting cells (APCs) [22,23]. The crossed NOD-SCID mice were found to have increased engraftment efficiency, making them a better model for studying human disease [24].

Around the same time, during the early 1990s, recombination activating gene 1 (*RAG-1*)-deficient mice were created, which lacked both arms of the adaptive immune system [25]. RAG-1 is essential for V(D)J recombination by initiating double-stranded DNA breaks during the early stages of B and T cell development [26]. RAG-1KO mice were invaluable to studying B and T cells in cancer and autoimmunity [27]. However, they are infrequently used as the background strain to generate humanized mice due to their intact myeloid compartments. The next major contribution to the field was the creation of common cytokine gamma-chain knockout mice in the late 1990s, which were found to have no NK cells and fewer functional B and T cells [28]. Additionally, the common gamma chain is a necessary component in IL-2, IL-4, IL-7, IL-9, IL-15, and IL-21 cytokine signaling pathways that are central to T cell differentiation, proliferation, and memory [29]. There were two approaches to knocking out the common gamma chain and crossing it to NOD-SCID mice. The first, conducted by Ito et al., was to truncate the intracellular signaling domain, which led to the protein being produced but unable to induce downstream signaling cascades [30]. The second, conducted by Shultz et al., was to introduce a null mutation in the gene so no protein was produced [31]. These are now identified as “NOG” (created by Ito et al.) and “NSG” (created by Shultz et al.). Both mouse lines were first successfully engrafted with HSCs, shown by higher cell numbers and a proliferative response when subjected to a pathogenic challenge [30,31]. Like SCID mice, RAG-1KO mice were crossed to the NOD/Lt and gamma-chain knockout mice to increase human cell engraftment, generating the “NRG” strain [32]. NRG mice were found to have a larger amount of T cell engraftment compared to NOD/SCID and NOD/RAG-1KO mice and similar proportions of B cells and myeloid cells [32]. Currently, both the NSG and NRG strains are commonly used to create humanized mice. More recently, “BRG” mice were created by crossing Rag2^−/−^ and gamma-chain knockout mice on a Balb/c background [33]. These background strains are more comprehensively summarized in Table 1.

More work is being conducted to add critical human factors to these mice, in addition to further depleting murine immune cells. This has included the generation of NSG-SGM3 mice (NOD.Cg-*Prkdc^scid^*Il2rg^tm1Wl^ Tg(CMV-IL3, CSF2, KITLG)1Eav/MloySzJ), which have human IL-3, stem cell factor, and GM-CSF and improved human myeloid cell development and engraftment [34]. As an extension of the BRG model, BRGSF mice (BALB/c *Rag2^tm1Fwa^Il2rγ^tm1Cgn^Sirpα^NOD^Flt3^tm1lrl^*) have a deletion of *Flt3*, which further reduces the murine myeloid compartment [35]. To address the issue of HLA restriction, mouse models have been created with human HLA genes. This includes DRAG (NOD.Cg-*Rag1^tm1Mom^Il2rg^tm1Wjl^* Tg(HLA-DRA,HLA-DRB1*0401)39-2Kito/ScasJ) mice and NSG-HLA-A2/HHD (NOD.Cg-*Prkdc^scid^Il2rg^tm1Wjl^* Tg(HLA-A/H2-D/B2M)1Dvs/SzJ) mice [36,37]. Lastly, two models have been created to improve human NK cell development using the addition of human cytokines, including IL-2-NOG transgenic (NOD.Cg-*Prkdc^scid^Il2rg^tm1Sug^*Tg (CMV-IL2)4-2Jic/Jic) and IL-15-NOG transgenic (NOD.Cg-*Prkdc^scid^IL-2Rrgc^m1Sug^* Tg(CMV-IL2/IL15)1-1Jic/JicTac) mice [38,39]. These strains have been used to study NK cell biology in vivo since these cytokines are critical for NK cell survival, proliferation, and function [38,39]. The continued development of immunodeficient mice by academic labs and commercial companies will impact the field and more faithfully replicate the human immune system in a small animal model.

## 3. Humanized Mouse Models

**Figure 1:**
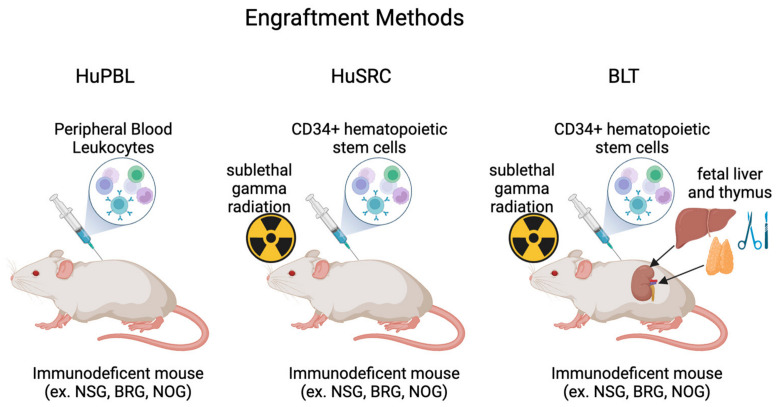
Schematic representation of the different engraftment methods routinely used to generate humanized mice. Created with BioRender.com.

Humanized mice are typically generated by engrafting human immune cells into immunodeficient mice [40]. Factors such as cell/mouse strain availability, cost, technical capacity, and experimental time frame play a role in determining what model best fits a particular experiment/research question. The most utilized background strains are NOG, NRG, NSG, and BRG, which vary in the type of endogenous murine cells and, hence, have varied extents of human cell proliferation/function post-engraftment [41]. Typical approaches to humanization involve engraftment with either (1) human peripheral blood leukocytes (Hu-PBLs), (2) human CD34+ cells (Hu-SRCs), or (3) human fetal bone marrow/liver/thymus (BLT) (Figure 1) [42]. These approaches have advantages and disadvantages, discussed in the subsequent sections and summarized in Table 2.

The first humanized mice, published by Mosier in the late 1980s, used Hu-PBLs to engraft SCID mice [21]. They tested two cell administration methods, i.e., intraperitoneal or intravenous injection, and determined that intraperitoneal was more effective in delivering increased human lymphocyte populations in the animal [21]. Subsequent work determined the optimal experimental conditions, including the number of cells injected and whether to subject mice to irradiation before transfer [43]. The background strain used when engrafting Hu-PBLs has evolved as immunodeficient mouse strains themselves have evolved, beginning with SCID mice, then NOD/SCID mice, and now NSG mice [44]. When engrafted into SCID mice, graft vs. host disease (GvHD) can occur after four to six weeks, rendering these mice useful for only short-term experiments [45]. Interestingly, NSG mice with additional defects in murine MHC1 and MHC2 can be used for extended experimental periods without the occurrence of GvHD [46]. Once in the recipient, reconstitution occurs quickly due to the lack of multilineage hematopoiesis (3–5 days) [13]. However, while PBLs lead to robust T cell engraftment/reconstitution, they have defective human myeloid cells and B cells, limiting their applications [47]. Additionally, the majority of human T cells in these mice are not naïve and instead have a memory or activated phenotype [48]. The Hu-PBL models are popular as the humanization technique is relatively easy and inexpensive. These mice are used extensively in virology research (including both HIV and EBV) [49,50] and cancer [51], as many viruses have human-specific tropism and are unable to infect mice [52]. Moreover, in the era of personalized medicine, Hu-PBL mouse models are also routinely used to examine patient-specific therapeutic responses [53,54].

The idea of engrafting immunodeficient mice with human hematopoietic cells to generate Hu-SRC mice began with the implantation of a fetal thymus, lymph node, and fetal liver cells on SCID mice [55]. This engraftment methodology successfully led to the presence of human CD4 T cells, CD8 T cells, and IgG in the periphery of the immunodeficient mice [55]. Next, Kamel-Reid and Dick used adult bone marrow cells, which led to human immune cell growth in the animal [56]. Since then, cell sources have been expanded to include human umbilical cord blood, adult bone marrow, fetal liver, and G-CSF-administered/mobilized PBMCs [30,31,57,58]. The choice of cell source is critical when setting up a humanized mouse model, as it impacts the function and capacity of the engrafted cells. Another factor that influences the human immune cell development is the age of the engrafted mice, as both newborn and adult mice can be engrafted with HSCs. This choice impacts the engraftment procedure, as adult mice receive cells administered intravenously or intrafemorally, while newborn mice are engrafted via intracardiac or intrahepatic injection [15]. Advantages of the Hu-SRC model include the lack of surgery, the occurrence of multilineage human immune hematopoiesis (leading to reconstitution and functional T cells, B cells, and myeloid cells), and the generation of primary human immune responses within the animal [31,57,59]. Hu-SRC mice also have several disadvantages. This hematopoiesis and full reconstitution of human immune cells can take up to 12 weeks, leading to extended experimental timelines compared to Hu-PBL mice [40]. Additionally, since T cells are educated in the mouse thymus (based on mouse MHC molecules), their activation via human HLA on human APCs is dysfunctional [60]. Typically, NSG and BRG background strains are used for generating Hu-SRC mice and are being utilized in cancer and infectious disease research [61,62,63].

To more fully recapitulate human immune cell development, the BLT model, which was developed in the early 2000s, included engraftment of human fetal bone marrow, liver, and thymus into irradiated NSG/SCID mice [64]. These mice were found to have increased longevity of human B cells, T cells, and DCs, as well as the presence of human IgM and IgG [64]. When challenged with both Epstein–Barr Virus (EBV) and toxic shock syndrome toxin-1 (TSST-1), BLT mice elicited a functional T cell response, shown via cytokine production [65]. Compared to the Hu-PBL and Hu-SRC models, the BLT model leads to the most comprehensive reconstitution of the human immune system [42]. Since this model is an extension of the Hu-SRC model, both involving the transfer of human CD34+ cells, some of the advantages of the BLT model are similar. This includes having multilineage human immune hematopoiesis and more accurate T cell education that is not HLA-restricted due to the presence of the human thymus [47]. By not having HLA restriction, BLT mice can develop a T and B cell repertoire that mimics human lymphocyte development [66]. However, there are notable disadvantages to the use of BLT mice, including a technically difficult and expensive engraftment procedure, as it requires surgery, sublethal irradiation, and the acquisition of human fetal cells. Additionally, animals can develop graft vs. host disease (GvHD) 20–22 weeks post-engraftment and are susceptible to lymphohistiocytosis, in which human macrophages target and kill murine erythrocytes, leading to increased proinflammatory cytokine production [67,68,69]. Active work using these BLT mice is being conducted within the HIV and cancer fields [70,71].

**Table 2:** Cell subsets in humanized mice. The table shows the advantages and disadvantages of each engraftment model, both generally and at a cell-specific level (including T cells, B cells, and NK cells).

Name	Cell Type	Advantages	Disadvantages	Reference
Hu-PBL		Easy and inexpensive technique, rapid reconstitution	GvHD after 4–6 weeks, no multi-lineage hematopoiesis	[44,46]
	B cells	-	Low levels of human B cells present, cannot mount primary response	[46]
	T cells	Robust T cell reconstitution	Uniform T cell activation due to MHC mismatch, generally not naïve cells	[49]
	NK cells	-	Largely absent	[46]
Hu-SRC		Multilineage hematopoiesis, no surgery required, primary immune responses, extended experimental timeframe (up to 12 weeks)	Requires sub-lethal irradiation	[31,40,58,60]
	B cells	Improved antigen-specific B cell responses, B cell maturation	Limited class switching due to low levels of IgG	[58]
	T cells	Robust T cell reconstitution	T cell education in the absence of human thymic epithelial cells (HLA mismatch)	[61]
	NK cells	Increased initial number and function of NK cells	Minimal long-term NK cell survival	[39]
Hu-BLT		Multilineage hematopoiesis, sustained immune cell lifespan	Requires sub-lethal irradiation, technically difficult, limited tissue availability	[48,65]
	B cells	Improved antigen-specific B cell response	Low levels of IgG and class switching	[65]
	T cells	Human thymic education of functional T cells (no HLA restriction)	-	[66]
	NK cells	Functional and can survive (only when engrafted in IL2rg deficient mice)	-	[41]

## 4. Applications in Bacterial Infections

**Figure 2:**
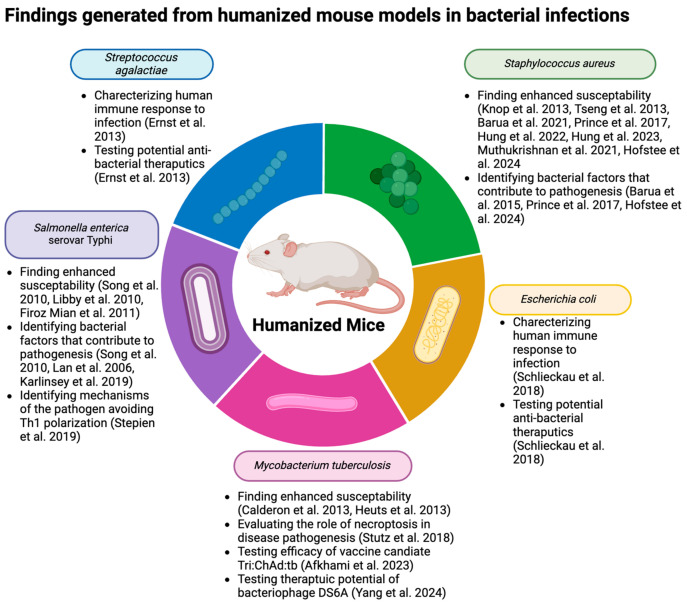
Schematic representation of key findings from humanizd mouse models of bacterial infections. References include citations [33,64,78,79,80,81,82,93,94,95,96,97,98,99,100,109,110,111,112,115,116]. Created with BioRender.com.

Humanized mice have been used to interrogate multiple bacterial infections, which has led to significant advances in our understanding of human disease during bacterial infections (Figure 2). The current review will outline how humanized mice were employed to understand Mycobacterium, Staphylococcus, Salmonella, Streptococcal, and *E. coli* infections. Generally, work began for each bacterium by characterizing enhanced susceptibility to disease and then interrogating disease-specific mechanisms of pathogenesis or potential therapeutics.

### 4.1. Tuberculosis

Tuberculosis (TB), a disease caused by *Mycobacterium tuberculosis (M. tuberculosis)*, is a significant problem to human health. A recent World Health Organization(WHO) report indicated that 10.6 million new individuals are infected with *M. tuberculosis* yearly [72]. Latent infections are also extremely prevalent and occur in approximately 1.7 billion people [73]. TB can also occur with comorbidities such as HIV or diabetes and is linked to enhanced mortality and morbidity [74]. Currently, the standard-of-care treatment for TB is a four-drug, six-month antibiotic regimen, which is curative [75]. A key feature of tuberculosis, lung granulomas, does not occur in mouse models due to their requirement for human CD4 T cells, making the disease difficult to model [76,77]. Therefore, the application of humanized mice in the context of tuberculosis has shed light on disease pathogenesis and led to insights regarding improved vaccines and therapeutics.

In 2013, two groups used humanized mouse models to investigate tuberculosis susceptibility in humanized mice. Calderon et al. characterized human immune cell effector capacity and then infected Hu-BLT-NSG mice with *M. tuberculosis* strain H37Rv and observed lung and liver pathology (including the presence of granulomas) with 250 colony-forming units (CFUs) of bacteria, a dose relevant to human disease [78]. This is a much lower dose than what has been used in conventional mice, which is up to 10^5^ CFUs [79]. They also observed human T cells at the site of infection using immunohistochemistry (IHC) [78]. Heuts et al. infected Hu-SRC-NSG mice with *M. tuberculosis* (Harlingen strain) and compared disease susceptibility between engrafted and non-engrafted mice [77]. The authors observed more lesions and necrotic tissue in both the liver and the lung of Hu-SRC-NSG mice [77]. Together, these studies formally established that humanized mice are a more clinically relevant small animal for tuberculosis.

More recently, humanized mice have been utilized to investigate the role of necroptosis in *M*. tuberculosis disease pathogenesis [80]. When the authors inhibited necroptosis before and during infection, there were no differences in bacterial burden or dissemination in the lung and the spleen in Hu-SRC-NSG mice [80]. To test the efficacy of a multivalent vaccine candidate Tri:ChAd:tb, a group challenged Hu-SRC-NSG mice with *M. tuberculosis* strain H37Rv and found decreased bacterial burden, reduced granulomatous lesions, and reduced gross pathology with immunized mice [81]. Yang et al. infected Hu-SRC-NSG-SGM3 mice with *M. tuberculosis* strain H37Rv to test the therapeutic potential of bacteriophage DS6A [82]. When treated with phage, mice had improved pulmonary function, less inflammation, less dissemination, and a strong human IgM response [82]. These studies show the potential for humanized mice to verify findings from traditional mouse models or test potential therapeutics within the context of tuberculosis.

### 4.2. Staphylococcal Infections

The Staphylococcus genus was first described by Sir Alexander Ogston in 1880 when he found the bacteria in a knee abscess [83]. Since then, over 40 species have been identified, with multiple staphylococcal species having roles in human homeostasis as commensals and/or human disease as pathogens [84]. *Staphylococcus aureus* (*S. aureus*) has been acknowledged to be a commensal and a pathogen [84]. As a commensal, it colonizes the skin and mucosal membranes, including the nose and throat in about 40% of healthy humans [85,86,87]. *S. aureus* can also manifest as a pathogen and infect over 50 human body sites, including skin, soft tissue, heart, lung, bone, and blood [88]. *S. aureus* is particularly a problem in hospitals, where it can become an opportunistic pathogen [89]. Complicating this matter is the increasing occurrence of antibiotic resistance and the spread of methicillin-resistant *S. aureus* (MRSA), leading to chronic infections due to a failure to achieve bacterial clearance [90]. *S. aureus* has been established to be a well-adapted human pathogen, shown by high tropism of many immunotoxins and virulence factors (including Panton–Valentine leucocidin (PVL), leucocidin AB (LukAB), and gamma hemolysins (HlgCB)) to human leukocyte receptors [91,92]. Therefore, using humanized mice instead of standard murine models can inform disease pathogenesis in humans and assist in developing potential therapeutics.

Many humanized mice models have been used to investigate different Staphylococcal infections. The first use of humanized mice was by Knop et al., where the group utilized Hu-SRC-NSG mice to evaluate susceptibility to PS80 (an *S. aureus* clinical isolate) peritoneal infection [93]. They found that humanized mice had decreased survival and higher bacterial burden than WT and non-engrafted NSG mice [93]. Additionally, they observed infection-induced T cell invasion, activation, and apoptosis within the humanized mice [93]. Next, Tseng et al. evaluated skin infection with CST5 (a community-associated MRSA strain) using a Hu-SRC-NSG model and found enhanced infection susceptibility compared to non-engrafted NSG mice [94]. Similarly, Barua et al. observed increased susceptibility to skin infection when an ST30 *S. aureus* strain was used in a Hu-SRC-NSG model [95]. Prince et al. utilized Hu-SRC-NSG to model *S. aureus* infections in the lung. Pulmonary infection with MRSA USA300 strain revealed a more severe infection phenotype and increased local human inflammatory response in the Hu-SRC-NSG mice lung than controls [96]. In both the skin and lung, the authors also identified *S. aureus*-specific factors that play a role in pathogenesis, such as PVL and vancomycin-resistance-associated regulator (*vraR*) [94,95,96]. By identifying the same important protein in multiple infection sites, we may be able to identify more conserved important determinants of *S. aureus* pathogenesis.

To investigate local acute intramuscular infection, Hung et al. used Hu-SRC-NSG mice infected with USA300 and found enhanced infection susceptibility, as demonstrated by higher local bacterial burden in acute infection (seven days post-infection), enhanced mortality, and increased occurrence of internal organ dissemination [97]. Interestingly, they correlated higher rates of human cell engraftment (measured as hCD45%/(hCD45+mCD45 using flow cytometry) with higher chances of mortality [97]. The authors followed up a year later with a study comparing these mice to the same model with Hu-SRC-NSG-SGM3 mice and found a more severe phenotype [98]. Specifically, they observed higher mortality, higher bacterial burden in internal organs and more human immune cells in the blood and spleen two days post-infection [98]. The authors also concluded that the NSG-SGM3 mice had more human T cells, B cells, and CD45+ cells in the blood compared to NSG mice, suggesting that they may be a better model for human disease [98].

*S. aureus* is known to cause chronic infections in the bone, which has been modeled in humanized mice. The first published study in 2021 used Hu-SRC-NSG mice infected with USA300, where researchers found increased infection susceptibility compared to WT and non-engrafted NSG mice [99]. Muthukrishnan et al. demonstrated that *S. aureus* induces a human immune response during osteomyelitis and that these mice have increased osteolysis, increased local and systemic bacterial burden, and more Staphylococcal Abscess Communities (SACs) at the site of bone infection compared to control animals [99]. Despite this severe infection phenotype, the authors observed proliferating CD3+Tbet+ cells adjacent to the infection site, suggesting that human T cell infiltration is insufficient for generating bacterial clearance in the bone [99]. More recently, Hofstee et al. used Hu-SRC-BRGSF mice infected with USA300 to evaluate the role of PVL on disease pathogenesis during acute infection [100]. They found that humanized mice infected with Δ*PVL* had less local and systemic bacterial burden and no SACs at three days post-infection [100]. This was linked to fewer HLA-DR+ monocytes/macrophages and fewer dead cells in the bone marrow during infection with the Δ*PVL* mutant. This suggested that PVL alters innate immune responses in this model. These studies have shown the capacity of humanized mice to study human immune responses to *S. aureus* to provide a more molecular understanding of host–pathogen interactions.

### 4.3. Salmonella

*Salmonella enterica* serovar Typhi (*S. Typhi*) causes typhoid fever, a febrile illness commonly caused by ingesting contaminated food or water [101]. A Centers for Disease Control and Prevention report estimated that there were 9.2 million typhoid fever cases and 110,000 deaths worldwide in 2019, showing the magnitude of this problem [102]. The occurrence of multidrug-resistant *S. Typhi* infection cases is on the rise, causing difficulties in treatment and necessitating further studies on disease pathogenesis, novel vaccine candidates, and therapeutic interventions [103,104]. However, *S. Typhi* infections in conventional mice do not lead to progressive infection, largely due to the presence of TLR11, making it difficult to interrogate disease mechanisms [105]. To combat this, *S. Typhimurium*, a distinct but closely related Salmonella strain, is routinely utilized in mice studies and leads to progressive infection; however, *S. Typhimurium* possesses different virulence factors than *S. Typhi*, which significantly affects disease pathogenesis in mice [106,107,108]. Due to these challenges, humanized mouse models have led to novel insights into how the human immune system contributes to disease pathogenesis and spurred innovations in therapies to combat *S. Typhi* infections.

In the early 2010s, three studies were published on the susceptibility of various humanized mouse models to *S. Typhi* infections. Libby et al. infected Hu-SRC-NSG mice with *S. Typhi* strain Ty2 via intraperitoneal injection and found significant mortality within 48–72 h and more severe infection phenotype than both wildtype (C57BL/6) and non-engrafted NSG mice [109]. The authors observed extensive hepatocellular injury, Kupffer cell swelling, splenic granulomas, and cell death in the spleen in Hu-SRC-NSG mice [109]. The group also observed significant induction of proinflammatory human cytokines IL-6, IFNγ, TNFα, and MCP-1 [109]. In the same year, Song et al. published a study where the authors infected Hu-SRC-BRG mice with *S. Typhi* strain ISP2825 intravenously for four weeks and saw increased dissemination to the spleen, liver, and gall bladder compared to non-engrafted controls [33]. They also observed the induction of human cytokines, including IL-10, TNFα, and MIP-1α at four weeks post-infection [33]. Utilizing this model, the authors also tested the impact of specific *S. Typhi* gene knockouts and found Δ*phoP*Δ*phoQ* had a lesser bacterial burden and Δ*DpltB* (a typhoid toxin subunit) had more [33]. Lastly, Mian et al. infected Hu-SRC-BRG mice with *S. Typhi* strain BKC 3233 intravenously and observed more severe weight loss and less survival than control non-engrafted mice at nine days post-infection [110]. They also quantified bacterial burden at two days post-infection and found more bacteria in the liver, spleen, bone marrow, and blood compared to controls [110]. These studies differed in their bacterial administration route, *S. Typhi* strains used, dosage, and mouse model, all of which would affect disease phenotype and infection severity. Nonetheless, these studies demonstrated that humanized mice have progressive infection when given *S. Typhi*.

More recently, humanized mice have been used to study specific disease pathogenesis mechanisms. Karlinsey et al. infected Hu-SRC-NSG mice with an *S. Typhi* transposon library to identify mutants critical for disease pathogenesis [111]. They found bacteria lacking Vi antigen (*vexA*) or enterobactin synthesis (*entA*) were outcompeted by WT, while SPI-2 (*ssrB*) and typhoid toxin (*cdtB*) were not needed for virulence. A follow-up study by Hamblin et al. investigated the role of *S. Typhi’s* type three secretion systems (T3SS-1 and T3SS-2) in virulence, first utilizing human macrophages and then using Hu-SRC-NSG mice to use as an in vivo confirmatory model [61]. Specifically, the authors infected the mice with an equal mixture of WT and isogenic single Δ*ssaV* mutant or double Δ*invA* Δ*ssaV* mutant and calculated the bacterial survival and competition index at five days post-infection. The authors observed that WT outcompeted both mutants in the spleen and the double mutant also outcompeted both WT and single mutant in the liver. These results suggest that both type three secretion systems are important for bacterial colonization in the humanized mouse model of typhoid fever. Most recently, Stapien et al. also followed up on in vitro work to investigate the role of Th1 polarization in chronic infection [112]. They utilized a Hu-SRC-NSG model and infected with *S. Typhi*, WT *S. Typhimurium*, or Δ*ssrB S. Typhimurium*, finding that, at day seven post-infection, WT. *S. Typhimurium* led to the highest induction of human IL-12 and IFN-γ. The authors concluded that *S*. *Typhi* can avoid Th1 polarization, leading to its ability to cause chronic infection. These studies demonstrated the potential of combining humanized mouse models with bacterial genetics to ask mechanistic questions regarding *S. Typhi* pathogenesis.

### 4.4. Sepsis/Other Bacterial Infections

Sepsis, the third major cause of neonatal death, is a systemic illness, caused by a disruption in host barrier integrity and microbial invasion into sterile parts of the human body, typically the blood, and a corresponding immune response [113,114]. This can be caused by a variety of pathogens, including group B Streptococcus species (ex. *Streptococcus agalactiae*), *Escherichia coli* (*E. coli*), *S. aureus*, *Enterococcus*, *Klebsiella pneumoniae*, and *Acinetobacter baumannii* [113]. Two studies utilized humanized mice to investigate neonatal sepsis, the first by Ernst et al. using *Streptococcus agalactiae* and the second by Schlieckau et al. using *E. coli.* Ernst et al. infected Hu-SRC-NSG mice with *Streptococcus agalactiae* strain ATCC 13813 intraperitoneally and evaluated the human immune response to infection [115]. The authors found an induction of CD33+ cells in the peripheral blood during infection and increased human immune cell infiltration in the peritoneal cavity over time (days 1, 3, and 7 post-infection). Additionally, they quantified an induction of human cytokine production at day one post-infection (including IFN-γ, IL-10, IL-1B, and IL-6). To test the usage of this model, the authors administered betamethasone (an anti-inflammatory corticosteroid) and indomethacin (a nonsteroidal anti-inflammatory drug), both given routinely during preterm birth, and observed that betamethasone reduced systemic bacterial clearance, leukocyte migration, and proinflammatory cytokine production. This study showed the capability of *Streptococcus agalactiae* to induce a human immune response and the potentially detrimental impacts of betamethasone. More work is warranted in investigating the combination of these standard-of-care treatments with antibiotics. Following this work, Schlieckau et al. infected Hu-SRC-NSG mice with *E. coli* intraperitoneally and performed the same outcome measures [116]. They looked at leukocyte populations in the peripheral blood and peritoneum and found a higher proportion of human CD14+ cells in both sites compared to uninfected controls. At day one post-infection, there were increases in human IL-6, IFN-γ, MCP-,1, and MIP-1β. Treatment with betamethasone and indomethacin led to no differences in bacterial load with either drug, but betamethasone did increase the proportion of CD45+ cells in the peripheral blood. The authors concluded that humanized mice could effectively model *E. coli* neonatal sepsis due to the similarities in the elicited human immune response. This may provide an avenue for testing potential therapeutics, as many therapies have shown effectiveness in conventional mice but failed human clinical trials [116]. These two studies show interesting similarities in disease progression regardless of the causative agent in humanized mouse models of neonatal sepsis. Interestingly, while bacterial infections, in general, were more severe in humanized mice, neonatal sepsis due to bacteria was less severe in these mice [115,116]. It is unknown what contributes to these differences. Utilizing this model to investigate additional sepsis-causing pathogens (ex. *Klebsiella pneumoniae* and *Enterococcus* species) may help reduce the burden of these infections.

## 5. Conclusions and Future Perspectives

Small animal models will never faithfully replicate human diseases in their entirety. Still, generating clinically relevant models of human disease is increasingly possible due to the development and advancement of humanized mouse models. Progress has been achieved in successfully modeling human-adapted bacterial pathogens in small animal humanized mouse models. However, there is much to be done to improve these models so that we can truly and fully recapitulate the human system.

The microbiome and viriome are known to impact the immune response by directly interacting with immune cells and regulating metabolism, and it has also been established that mice and humans have different microbiomes and viriomes [117,118]. Therefore, developing humanized mice with human microbiomes and viriomes may help mimic human immune system priming and tolerance regulation. To date, all bacterial infection studies utilizing humanized mice have predominantly used CD34+ stem cells or fetal liver cells as the source of human immunity. The use of BLT models, which more comprehensively mimic human immune development, should be adapted more by researchers. Humanized mouse models with ectopic human organs should also be adapted for studying human tissue-specific pathology [119]. A case in point is the study by Wahl et al., in which the authors developed a humanized lung mouse model and showed permissive infection of human-specific viruses, including MERS-CoV, ZIKV, and HCMV [119]. Finally, work using humanized mice may spur the development of personalized medicine. They have been utilized to evaluate therapeutic options in cancer patients and could be applied to test antibiotics for bacterial infections. Additionally, due to the heterogeneity of bacterial strains causing severe infections, understanding the capacity of a patient’s in vivo immune response to a pathogen may provide insight into what therapies would be helpful. In conclusion, humanized mouse models have immensely improved our understanding of host–pathogen interactions, and their continued development is critical.

## Figures and Tables

**Table 1:** Immunodeficient murine model background strains. The table shows different immunodeficient mouse strains and their phenotypes.

Name	Strain	Phenotype	Reference
NOD-SCID	NOD.Cg-*Prkdc^scid^J*	T and B cell deficient due to *Prkdc* mutation	[22,24]
NOG	NOD.Cg-*Prkdc^scid^Il2rg^tm1Sug^/ShiJic*	T and B cell deficient due to *Prkdc* mutation, defective NKs and APCs due to truncated IL2rg	[30]
NSG	NOD.Cg-*PrkdcˢᶜᶦᵈIl2rgᵗᵐ^1^ᵂʲˡ/SzJ*	T and B cell deficient due to *Prkdc* mutation, defective NKs and APCs due to IL2rg knockout	[31]
NRG	NOD.Cg-*Rag1ᵗᵐ^1^ᴹᵒᵐIl2rgᵗᵐ^1^ᵂʲˡ^c^*	T and B cell deficient due to *Rag1* mutation, defective NKs and APCs due to *IL2rg* knockout	[32]
BRG	C;129S4-*Rag2^tm1.1Flv^ Il2rg^tm1.1Flv^/J*	T and B cell deficient due to *Rag2* mutation, defective NKs and APCs due to *IL2rg* knockout	[33]
